# The comparative cost-effectiveness of colorectal cancer screening using faecal immunochemical test vs. colonoscopy

**DOI:** 10.1038/srep13568

**Published:** 2015-09-04

**Authors:** Martin CS Wong, Jessica YL Ching, Victor CW Chan, Joseph JY Sung

**Affiliations:** 1 Institute of Digestive Disease, Chinese University of Hong Kong, Shatin, Hong Kong, HKSAR, China; 2 School of Public Health and Primary Care, Chinese University of Hong Kong, Shatin, Hong Kong, HKSAR, China

## Abstract

Faecal immunochemical tests (FITs) and colonoscopy are two common screening tools for colorectal cancer(CRC). Most cost-effectiveness studies focused on survival as the outcome, and were based on modeling techniques instead of real world observational data. This study evaluated the cost-effectiveness of these two tests to detect colorectal neoplastic lesions based on data from a 5-year community screening service. The incremental cost-effectiveness ratio (ICER) was assessed based on the detection rates of neoplastic lesions, and costs including screening compliance, polypectomy, colonoscopy complications, and staging of CRC detected. A total of 5,863 patients received yearly FIT and 4,869 received colonoscopy. Compared with FIT, colonoscopy detected notably more adenomas (23.6% vs. 1.6%) and advanced lesions or cancer (4.2% vs. 1.2%). Using FIT as control, the ICER of screening colonoscopy in detecting adenoma, advanced adenoma, CRC and a composite endpoint of either advanced adenoma or stage I CRC was US$3,489, US$27,962, US$922,762 and US$23,981 respectively. The respective ICER was US$3,597, US$439,513, -US$2,765,876 and US$32,297 among lower-risk subjects; whilst the corresponding figure was US$3,153, US$14,852, US$184,162 and US$13,919 among higher-risk subjects. When compared to FIT, colonoscopy is considered cost-effective for screening adenoma, advanced neoplasia, and a composite endpoint of advanced neoplasia or stage I CRC.

Colorectal cancer (CRC) is a leading cause of morbidity and mortality worldwide^1^. It is the second most common cancer in men and third in women, comprising of more than 10% of all malignancies and 8% of all cancer deaths globally^1^. In the past few decades, many Asia Pacific countries like Japan, China, Korea, Singapore and Hong Kong have experienced a two- to three-fold rise in new CRC cases, which became increasingly comparable with the incidence figures in Western countries^2^. In Hong Kong, CRC ranks the second among the most common malignancies, and is also the second commonest cause of cancer-specific deaths^2^. The age-standardized incidence rates per 100,000 persons in 2010 were 47.0 and 30.1 for men and women, respectively^2^. The direct medical cost for the care of colorectal neoplasia was estimated at US$45,115 for stage IV CRC in the initial year of care, leading to a substantial public health burden to the healthcare system^3^.

It has been shown by randomized trials that CRC screening using Faecal Occult Blood Tests (FOBTs) and colonoscopy could effectively reduce cancer mortality by 33% and 56%, respectively^4^,^5^. Guidelines from authoritative societies in the US, the European nations and the Asia Pacific Working Group on CRC^6^ recommended screening among average-risk individuals aged 50–75 years. FOBTs and colonoscopy are amongst the most commonly used screening tools. However, national screening programmes for CRC are still under development in many Asia Pacific countries, and financial burden incurred on the healthcare system has been attributed as a major hesitancy for their implementation. Concrete evidence on the cost-effectiveness of the different screening modalities will need to be elucidated.

Previous studies on cost-effectiveness analysis comparing CRC screening modalities were few, and gave inconclusive recommendations. Also, these analyses relied heavily on assumptions of compliance and costs of procedures in the Western countries^7^,^8^,^9^,^10^,^11^, and the findings were based on decision analysis using computer software like Markov modeling^7^,^8^,^9^,^11^, or published estimates of cost and effectiveness of CRC screening strategies^10^. Evidence built on clinical transition probabilities may not be too convincing to policy-makers in making decisions regarding design and implementation of CRC screening programmes at a population level. As highlighted by Theuer and colleagues^12^, the cost-effectiveness of CRC screening programmes is largely determined by racial and ethnic patterns of CRC, as well as incidence rates of CRC across different races^12^.

Whereas colonoscopy has been regarded as a gold-standard diagnostic test for CRC, our previous study on the diagnostic accuracy of FIT among 5,339 Chinese asymptomatic screening participants showed that the overall sensitivity, specificity, Positive Predictive Value (PPV) and Negative Predictive Value (NPV) for advanced neoplasia or CRC was 26.2%, 93.3%, 18.3% and 95.7%, respectively^13^. Older individuals aged 66–70 years had higher sensitivity and PPV but lower specificity and NPV. Male subjects had slightly higher PPV but lower NPV. This study aims to compare the cost-effectiveness of Faecal Immunochemical Tests (FIT) vs. colonoscopy in the detection of various forms of colorectal neoplasia in a large cohort of Chinese screening participants. We based on “real-life” screening practice data to compare the two screening modalities. We tested the *a priori* hypothesis that there exists a substantial difference in the cost-effectiveness between these two competing strategies. The study included a cost analysis from a large cohort of CRC screening participants in Hong Kong (Table 1).

## Results

### Participant Characteristics

From a total of 10,732 screening participants, 5,863 joined the annual FIT scheme and 4,869 chose colonoscopy. The screening-related costs and treatment-related costs for both groups were shown in Table 2, whereas their characteristics were shown in Table 3. Subjects in the colonoscopy group were younger, more were male, and higher proportions reported family history of CRC with their first degree relatives affected (Table 3). The compliance rates with yearly FIT were 97.3%, 82.8%, 84.6% and 77.7%, respectively, in the first four years of follow-up. Among those who chose colonoscopy, 90.7% attended for the procedure (n = 4,418); whereas 89.8% of participants in the FIT group who had positive faecal test results completed colonoscopy (n = 343). The positivity rates of FIT in the first four years were 5.2%, 1.0%, 0.9% and 0.9%, respectively. Among those who received colonoscopy, there were 57.1% and 41.8% of participants who had polypectomy done in the FIT and colonoscopy groups, respectively. The adverse event rate of the FIT group was 2.9% and that of the colonoscopy group was 0.82%. In our database capturing the screening modalities undertaken, all compliant subjects assigned FIT underwent FIT in our centre, and only those FIT positive individuals received colonoscopy. Subjects who defaulted FITs did not receive any other CRC screening procedures in the public healthcare sector, as far as we are aware of. Similarly, all compliant subjects assigned colonoscopy attended colonoscopy, and those defaulted did not receive any screening colonoscopy in the public healthcare sector to the best of our knowledge.

### Expenditure and colonoscopic yield according to the screening modality

The yearly FIT scheme was much less expensive than the colonoscopy scheme (US$1.47 million vs. US$5.17 million; Table 4). The majority of the costs incurred in the yearly FIT scheme included the FIT kits (52.7%), whilst most of the expenses for those who chose colonoscopy were attributed to colonoscopic procedures, costs required to manage its complications, and consultation fees 95.1%). Among study participants who chose FITs, the proportions of those found to have adenoma, advanced neoplasia and cancer were 1.6%, 1.0% and 0.2%, respectively. The corresponding proportions among those who received colonoscopy were 23.6%, 3.9% and 0.3%, respectively (Table 5).

### Comparative Cost-Effectiveness between the FIT and colonoscopy schemes

The total screening costs for applying annual FIT scheme and the colonoscopy scheme were US$1.23 million and US$4.43 million, respectively (Table 6). The cost of detecting one colorectal adenoma using FIT was higher than colonoscopy (US$13,176 vs. US$4,271). However, use of FIT as a primary screening tool was found to be cheaper than colonoscopy in detecting one advanced neoplasia (US$21,882 vs. US$26,151), CRC (US$0.123 million vs. US$0.357 million), or a composite endpoint of either advanced neoplasia or stage I CRC (US$21,521 vs. US$23,294). Repeating the analysis in moderate-risk individuals and applying a yearly inflation rate of 3%, separately, leads to a similar conclusion. However, when only high-risk individuals were included in the analysis, FIT was found to be more expensive to detect one adenoma (US$9,959 vs. US$3,590), one advanced neoplasia (US$19,918 vs. US$15,555), and one advanced neoplasia or stage I CRC (US$19,708 vs. US$14,720). The cost of detecting one CRC using FIT was lower than colonoscopy (US$128,306 vs. US$541,761) (Table 6).

Using FIT scheme as a control, the incremental Cost-Effectiveness Ratio of screening colonoscopy was US$3,489, US$27,962, US$922,762 and US$23,981 to detect one adenoma, advanced neoplasia, CRC, and a composite endpoint of advanced neoplasia or stage I CRC respectively. The respective incremental cost effectiveness ratio (ICER) of screening colonoscopy to detect these lesions in lower-risk subjects was US$3,597, US$39,513, -US$2,765,876 (dominated) and US$32,297 for lower-risk subjects. The corresponding ICER was US$3,153, US$14,852, US$184,162 and US$13,919 for high-risk individuals (Table 6). Colonoscopy is considered cost-effective for screening adenoma, advanced neoplasia, and a composite endpoint of advanced neoplasia or stage I CRC, but not CRC alone.

## Discussion

### Major Findings and Implications to Screening Policy

These findings suggested that FIT, if tested positive, followed by colonoscopy is a cost-effective strategy to screen for both advanced neoplasia and CRC in the general population and those with lower risks for CRC. Colonoscopy is cost-effective to detect adenoma, advanced neoplasia and stage I CRC among higher risk subjects. The fact that FIT is cost-effective to detect advanced lesions in our cohort is coherent with the purpose of FIT to detect malignancy in screening programmes. Nevertheless, in screening practices where colorectal neoplasia is primarily targeted, or where higher risk subjects were screening participants, colonoscopy as a primary screening tool is preferred. People having adenomas are at an increased risk for developing metachronous adenomas or cancer when compared with those without adenomas, and it is widely recognized that detection and removal of adenomas can prevent cancers and reduce mortality^14^. The choice of screening modality for population-based CRC screening should therefore depend on the major objectives of the screening programme (detection of adenomas vs. advanced neoplasia) in order to optimize cost-effective screening practices. The population being screened is also a considering factor i.e. higher risk subjects could be prioritized to receive colonoscopy. The findings from this study provide useful references for policy-makers to make budgetary planning and consolidate programme implementation at a population level. In resource-deprived regions and countries where colonoscopic capacities were limited, FIT represents a potentially viable, cost-effective option as a screening modality for population-based CRC screening. It should be noted, however, that those who received FIT had higher adverse event rate in terms of bleeding. This may be due to the higher diagnostic yield of polyps among the FIT group than the colonoscopy group (57.1% vs. 41.8%)—thus increasing the risk of post-polypectomy bleeding.

### Relationship to existing literature

There were a few original studies which compared the cost-effectiveness of FIT vs. colonoscopy as one of their primary objectives^7^,^8^,^9^,^10^,^11^,^15^,^16^,^17^,^18^,^19^,^20^,^21^. Some analyses reported more favorable cost-effectiveness profiles of faecal tests^9^,^11^,^17^,^18^,^19^,^20^, whereas others concluded that colonoscopy was superior^7^,^8^,^15^,^16^. Three studies recommended, respectively, double contrast enema^10^; use of unrehydrated FOBT followed by sigmoidoscopy^9^; and hybrid strategies which utilize annual or biennial FIT screening for people aged 50–65 years followed by a single colonoscopy when they were 66 years old^21^. Eleven out of these twelve studies used Markov modeling, mathematical predictions and dynamic decision analytic framework, which heavily rely on assumptive estimates. Sekiguchi and colleagues^20^ examined the cost-effectiveness of FIT and colonoscopy in the Japanese nationwide survey of CRC screening, but retrospective data were used. Our study is therefore the first evaluation performed in the Asia Pacific region utilizing data from “real-life” screening practices—which prospectively recruited screening participants over a four year period. This is also the first study of this large scale which compared FIT and colonoscopy in the community setting—whilst studies conducted in the Asia Pacific regions are scarce. The present findings might not be easily compared with those from existing literature as we have used detection of colorectal neoplasia as the primary outcome of interests, instead of life years saved as used in most studies.

### Study Limitations

This study has several limitations. Firstly, the screening participants were self-referred and they were given a choice of their preferred screening modality. Critics might argue that the two screening strategies may not be directly comparable, given differences in the baseline characteristics and subsequent screening behavior. However, it is the objective of this study to compare the real-life differences in cost-effectiveness between the two screening tests, and randomization may introduce artificiality and hinder its external validity. In addition, we evaluated the detection rates of colorectal lesions as the outcome because of the short period of observation. The cohort could be followed-up for longer-term to capture cost per Quality-Adjusted Life Years (QALYs), which could represent a more meaningful outcome of interest. Since colonoscopy is a 10-year strategy, this interim analysis should preferably be extended for optimal comparison—i.e. between yearly FIT for 10 years and one time colonoscopy. Besides, since this pragmatic study aims to reflect the reality in population-based screening practices but not interventional settings, we calculated the incremental cost-effectiveness ratios from two different samples. Also, other screening modalities like flexible sigmoidoscopy, capsule endoscopy, faecal DNA tests and CT colonography have not been evaluated as they are less commonly used as screening tools at the population level. Furthermore, it should be addressed that there still exists possibilities that those who had negative FIT results might have undiagnosed colorectal lesions, and these have not been taken into account in the present analysis. Our study did not include indirect costs, like transportation costs to hospitals and productivity lost due to absence from work.

In summary, this analysis showed that FIT offers a cost-effective way of screening and detecting colorectal advanced lesions and CRC, and that colonoscopy is cost-effective among higher-risk subjects. Nevertheless, due to the lack of an accepted standard “cost per lesion detected” above which the society is unwilling to pay, it is difficult to definitively state superiority of one strategy over another without referencing a standard value above which a strategy is deemed “not cost-effective”. Health policy-makers should consider allocation of more resources for FITs as the screening modality for CRC for the general public, and this screening tool should be assigned a high priority especially in resource deprived regions. Future studies should evaluate the comparative performances of the various FITs available in the market, as well as other screening modalities like flexible sigmoidoscopy and capsule endoscopy—where their use in the community may optimize screening yield in the most cost-saving manner. Since cost-effectiveness ratios are also susceptible to changes according to different population subgroups, future evaluations should explore whether screening for subjects of different ethnicities in the community may yield different comparative findings between the various screening modalities.

## Methods

### Setting

Detailed descriptions of the screening practice have been presented elsewhere^12^,^22^,^23^,^24^,^25^,^26^. Briefly, the study utilized data from a bowel cancer screening centre, which was established in Hong Kong in 2008. The centre invited free CRC screening for eligible asymptomatic Hong Kong residents aged 50-70 years via media announcements. The CRC screening service provided was accessible to all Hong Kong residents. Data upon screening recruitment and outcomes between 2008 and 2012 were collected. Informed consent was obtained from all subjects. All experiments were performed in accordance with the Declaration of Helsinki. The study protocols were approved by the Clinical Research Ethics Committee of the Chinese University of Hong Kong (protocol CRE-2008.404).

### Study Design

Self-referred screening participants aged between 50 and 70 years were prospectively recruited for CRC screening. Registrations could be via telephone, fax, email, or walk-in.

### Participant recruitment

Subjects were eligible for the screening service if they: (i) were aged between 50 and 70 years; (ii) had no existing or previous symptoms suggestive of CRC such as haematochezia, malena, anorexia, or a change in bowel habits in the past four weeks, or weight loss of greater than 5 kg in the past six months; and (iii) had not undergone any CRC screening tests in the past five years. Exclusion criteria consist of having a personal history of CRC, colonic adenoma, diverticular disease, inflammatory bowel disease, prosthetic heart valve, or vascular graft surgery. Those with medical conditions which are recognized as contraindications for colonoscopy, like the use of double antiplatelets and cardiopulmonary insufficiency, were excluded. A team of trained staff in the centre examined the eligibility of each participant by a standardized checklist.

Eligible participants subsequently completed a self-administered questionnaire, including demographic details on their age, gender, family history of CRC, smoking status, drinking habits, previous medical history and long-term medication use. The completeness of questionnaires was further checked by trained volunteers, who also assisted survey completion for illiterate participants by reading the questions word-by-word. All participants attended an educational session on CRC by trained professionals, delivered by a standard video followed by health talks. The video included the following aspects of CRC: epidemiology, natural history, risk factors, clinical features, importance of regular screening, and procedures of the FIT and colonoscopy. The potential benefits and risks of FIT and colonoscopy were further elaborated by the speakers. All colleagues and volunteers involved were trained by a team of gastroenterologists, family physicians and public health professionals before the programme. The talks were delivered in a standardized manner with both FIT and colonoscopy being presented in a non-preferential manner. Each session lasted for approximately one-and-a-half hour with a maximum audience size of 30 persons. The participants were given a choice between yearly FIT for up to four years (two kits for the first 3 years and one kit for the fourth year), or a direct colonoscopy for CRC screening. One qualitative FIT (El Monte, CA, USA) was used for all subjects who chose faecal tests. It obviated the need for dietary restriction before the tests. Both options were free-of-charge, and so were the colonoscopies arranged for those who had positive FIT results. All participants were encouraged to make their own choice without interacting with other participants. The present study included all subjects who have undergone screening between 2008 and 2012.

### Outcome Variables and Cost Items

This cost-effectiveness analysis was based on the cost items from actual costs incurred in the programme^11^,^27^, and the items were shown in Table 1. The cost items were presented using the concept of Net Present Value (NPV) which takes a 3% discount rate per year. The major outcome variables include the costs required to detect one adenoma, advanced neoplasia and CRC, respectively. Advanced neoplasia is defined as any colorectal adenoma which had a size of ≥10 mm in diameter, high-grade dysplasia, villous or tubulovillous histologic characteristics, or any combination thereof, but does not include CRC. The present analysis included screening-related costs only for subjects who received FIT and colonoscopy, respectively, and subjects who received FIT vs. colonoscopy and found to have CRC were in different stages (i.e. subjects who received colonoscopy tend to have CRC detected at earlier stage (proportion of being diagnosed with Stage I, II, III and IV was 71.4%, 14.3%, 14.3% and 0%, respectively. The corresponding figures for FIT were 50%, 30%, 10%, 10%). The present analysis included the cost of treatment in the denominator (effectiveness) rather than the numerator (screening related cost), since the current difference in the treatment cost between the two groups is due to the variation in numbers of patients who received diagnosis of colorectal cancer.

Hong Kong has a mixed medical economy^28^ and the total annual expenditure on health and health care amounted to 4.9% of the gross domestic product (GDP) in 2008^29^. The per capita GDP of Hong Kong was around US$4,430. The healthcare system consists of the public and private sectors, and the former was mostly funded by the government general tax revenue (~95%). The public fee structure was heavily subsidized with minimal charges to residents utilizing healthcare services in the public sector (one specialist out-patient visit costs around US$12.9 inclusive of colonoscopy, medications and physician consultation. The public system offers universal coverage with 9–95% market share for inpatients (bed-days). Therefore, most individuals who received colonoscopy in the public follow the same fee schedule, except for some special groups like civil servants and those covered by social security allowances, where fee exemption might apply. The present cost evaluation was conducted taking reference from a previous publication^11^, which was based on the Government gazette fees^30^. The gazette listed the standardized fees payable for clinical services provided by the public healthcare, and the fees reflect the costs encountered for every residents in Hong Kong. We converted the cost from Hong Kong dollars to US dollars by the exchange rate in year 2008, and adopted an inflation rate of 3% per year to account for the differences in costs over time. The costs of FIT and colonoscopy procedure; consultation fee; expenditure to manage the adverse events of colonoscopy (bleeding, polypectomy, perforation); treatment costs for various stages of CRC; compliance with yearly FIT and colonoscopy; and FIT positivity rates were used in the analysis. The polypectomy included pathology charges. The additional costs included in the analysis take into account the number of polyps. In patients who were diagnosed as having CRC, the cost of investigation and staging (including the number of CT and PET scan), cost of cancer treatment (including chemotherapy and surgery) and cost of hospital admission (including labour cost for daily hospital care and disposable instruments) were built in. We reported the actual costs and detection rates of neoplasia among the participants of the CRC screening program, and the other extrapolated the findings to the entire population in Hong Kong assuming 100% adherence was obtained.

### Statistical Analyses

In this study, detection of colorectal lesions was regarded as a measure of effectiveness, and it is superior to detect more lesions when the different strategies were evaluated. The numerator for the ICER was the difference in direct costs between the two screening methods, and the denominator was the difference in the number of colorectal lesions detected. The time window to include any screening cost is from the starting point of the screening when the practitioners delivered services to the end point of screening when the CRC was found or when the test was negative. Since the individuals receiving FIT vs. colonoscopy had different risks for colorectal neoplasia, the Asia Pacific Colorectal Screening (APCS) scoring system based on age, gender, family history and smoking^31^ was used to stratify the subjects into the lower risk (scores for average risk: 0–1; moderate risk: 2–3) and higher risk (scores: 4–7) groups. As no subjects had APCS scores falling into the range of average risk (0–1), we compared the cost-effectiveness between the moderate risk and high risk subjects. The above analyses were repeated again according to these two risk tiers.

## Additional Information

**How to cite this article**: Wong, M. C.S. *et al.* The comparative cost-effectiveness of colorectal cancer screening using faecal immunochemical test vs. colonoscopy. *Sci. Rep.* **5**, 13568; doi: 10.1038/srep13568 (2015).

## Figures and Tables

**Table 1:** Costs items based on different screening scheme and treatment methods.

Cost item	Baseline value (US$)
One kit of FIT	26
Colonoscopy	967
Consultation fee	97
Bleeding	3,340
Polypectomy	194
Perforation	10,856
Treatment for the stage I or II of CRC	16,654
CT scan	516
PET scan	1,806
Colorectal surgery	7,192
Consultation fees (9 days)	2,612
Hospital charges (9 days)	4,528
Treatment for the stage III of CRC	27,489
CT scan	516
PET scan	1,806
Colorectal surgery	7,192
Consultation fees for 9 days	2,612
Hospital charges for 9 days	4,528
Chemotherapy: FOLFOX for 6 months	10,835
Treatment for the stage IV of CRC	72,193
CT scan	516
PET scan	1,806
Colorectal surgery	7,192
Consultation fees for 9 days (up to 30 days)	2,612
Hospital charges 9 days (up to 30 days)	4,528
Chemotherapy: FOLFOX and Avastin for 10 months	49,018

FIT: Faecal Immunochemical Tests; CRC: Colorectal Cancer.

**Table 2:** The ratio of screening-related costs vs. treatment-related costs among subjects who received FIT vs. colonoscopy.

	FIT	Colonoscopy
Overall		
Screening related cost	1,225,369	4,916,415
Treatment cost	244,494	254,826
Screening related cost/Treatment cost	5.0	19.3
Moderate Risk		
Screening related cost	1,026,448	3,792,324
Cost of treatment	151,001	116,578
Screening related cost/Treatment cost	6.8	32.5
High Risk		
Screening related cost	199,184	1,119,993
Cost of treatment	93,900	138,248
Screening related cost/Treatment cost	2.1	8.1

The risk refers to that calculated from the Asia Pacific Colorectal Screening (APCS) scoring system (reference 32). All costs were presented in US dollars.

**Table 3:** Baseline characteristics of the screening schemes.

	Annual FIT scheme (N = 5,863)	Colonoscopy scheme (N = 4,869)	p-value
**Age (years; mean [SD])**	57.88 (5.26)	57.03 (4.87)	<0.001
**Male gender (No./%)**	2,422 (41.3)	2,303 (47.30)	<0.001
**Body Mass Index (kg/m**^**2**^**; mean [SD])**	23.56 (3.25)	23.47 (3.16)	0.189
**Family history of CRC (No./%)**	625 (10.7)	705 (14.5)	<0.001
**Smokers (No./%)**	314 (5.4)	423 (8.7)	<0.001
**Diabetes Mellitus (No./%)**	470 (8.0)	316 (7.4)	0.245
**Hypertension (No./%)**	1,506 (25.7)	1,097 (22.5)	<0.001
**IHD / Heart Disease, n (%)**	100 (1.7)	86 (1.8)	0.811
**COAD, n (%)**	56 (1.0)	37 (0.8)	0.277
**Stroke, n (%)**	57 (1.0)	38 (0.8)	0.291
**Cirrhosis, n (%)**	7 (0.1)	8 (0.2)	0.535
**GERD, n (%)**	256 (4.4)	267 (5.5)	0.007
**1**^**st**^ **year compliance rate**	97.3%	—	
**2**^**nd**^ **year compliance rate**	82.8%	—	
**3**^**rd**^ **year compliance rate**	84.6%	—	
**4**^**th**^ **year compliance rate**	77.7%	—	
**1**^**st**^ **year FIT positivity rate**	5.2%	—	
**2**^**nd**^ **year FIT positivity rate**	1.0%	—	
**3**^**rd**^ **year FIT positivity rate**	0.9%	—	
**4**^**th**^ **year FIT positivity rate**	0.9%	—	
**Compliance to colonoscopy**	89.8%	90.7%	0.120
**Among subjects having colonoscopy:**	(N = 343)	(N = 4,418)	
Colonoscopy with polypectomy	57.1%	41.8%	<0.001
Bleeding rate	2.9%	0.8%	<0.001
Perforation rate	0%	0.02%	N/A
**Staging of CRC at diagnosis**	(N = 10)	(N = 14)	
I	50%	71.4%	0.403
II	30%	14.3%	0.615
III	10%	14.3%	1.000
IV	10%	0%	0.417

IHD Ischaemic Heart Disease; COAD: Chronic Obstructive Airway Disease; GERD: Gastro-Esophageal Reflux Disease; CRC: Colorectal Cancer.

**Table 4:** Total costs (US$) expended in each scheme (N = 10,732).

	Annual FIT scheme (N = 5,863)	Colonoscopy scheme (N = 4,869)
1^st^ year FIT	296,660	—
2^nd^ year FIT	225,986	—
3^rd^ year FIT	183,841	—
4^th^ year FIT	68,728	—
**Subtotal**	**US$775,215** (52.7%)	—
Consultation fee + Colonoscopy	379,296	4,433,875
Polypectomy	39,304	358,124
Bleeding	31,555	113,560
Perforation	0	10,856
**Subtotal**	**US$450,154** (30.6%)	**US$4,916,415** (95.1%)
Treatment for CRC Stage 1	87,410	166,540
Treatment for CRC Stage 2	52,446	33,308
Treatment for CRC Stage 3	28,856	54,978
Treatment for CRC Stage 4	5,782	0
**Subtotal**	**US$,244,494** (16.6%)	**US$254,826** (4.9%)
**Total**	**US$1,469,863** (100%)	**US$5,171,241** (100%)

FIT: Faecal Immunochemical Tests; CRC: Colorectal Cancer.

**Table 5:** Colonoscopy findings among screening participants.

	Annual FIT scheme	Colonoscopy scheme
**Enrollment**	**5,863**	**4,869**
Colonoscopy findings		
Adenoma	93 (1.6%)	1,151 (23.6%)
Advanced neoplasia	56 (1.0%)	188 (3.9%)
CRC	10 (0.2%)	14 (0.3%)

FIT: Faecal Immunochemical Tests; CRC: Colorectal Cancer.

**Table 6:** Cost to detect one colorectal neoplasia.

	Annual FIT scheme (N = 5,863)		Colonoscopy scheme (N = 4,869)		ICER with FIT as control
All					
Findings	**N**	**Cost per finding**	**N**	**Cost per finding**	
Screening Cost	US$1,225,369		US$4,433,875		
Adenoma	93	13,176	1,151	4,271	3,489
AN	56	21,882	188	26,151	27,962
CRC	10	122,537	14	357,172	922,762
AN + CRC Stage 1	61	21,521	218	23,294	23,981
Moderate Risk	**(N** = **4,940)**		**(N** = **3,805)**		
Screening Cost	US$1,026,448		US$3,792.324		
Adenoma	70	14,664	839	4,520	3,597
AN	46	22,314	116	32,692	39,513
CRC	8	128,306	7	541,761	Dominated by FIT
AN + CRC Stage 1	50	21,925	137	28,514	32,297
High Risk	**(N** = **923)**		**(N** = **1,064)**		
Screening Cost	US$199,184		US$1,119,993		
Adenoma	20	9,959	312	3,590	3,153
AN	10	19,918	72	15,555	14,852
CRC	2	99,592	7	159,999	184,162
AN + CRC Stage 1	11	19,708	79	14,720	13,919

FIT: Faecal Immunochemical Tests; CRC: Colorectal Cancer; ICER: Incremental Cost-Effectiveness Ratio; AN: advanced neoplasia.
